# An open-source and easily replicable hardware for Electrical Impedance Tomography

**DOI:** 10.1016/j.ohx.2022.e00278

**Published:** 2022-02-10

**Authors:** B. Brazey, Y. Haddab, N. Zemiti, F. Mailly, P. Nouet

**Affiliations:** a LIRMM, Univ Montpellier, CNRS, Montpellier, France

**Keywords:** Electrical Impedance Tomography, EIT, Hardware, Open-source, Replicable

## Abstract

Electrical Impedance Tomography (EIT) is a powerful imaging tool for investigating electrical properties of tissues such as that of human bodies. The cheap, harmless and portable nature of this tool has made EIT a popular choice in many biomedical applications. However, performing EIT requires strong development at both hardware and software levels. In particular, performing in-lab experiences remains a challenge due to the cost of commercially available devices or the complexity of systems proposed in scientific literature. In this paper, an efficient and easily replicable EIT hardware is presented. This hardware was developed with the objective of making EIT accessible to as many people as possible. It has been designed for operating frequencies between 1 kHz and 50 kHz, and can be used for in-lab validation of proof of concept. Special care has been paid to the choice of components in order to optimize the performance versus cost ratio. Also, the overall footprint has been reduced by using recent and up-to-date integrated circuits. In particular, the use of a lock-in amplifier is a compact solution that allows both narrow-band filtering of the signal and provides an easily quantifiable DC signal at the output. Circuit schematics as well as manufacturing files are shared so that understanding, replication and improvement of circuits are facilitated. Fabrication and usage procedures are given as well. At last, the proposed hardware is experimentally tested and validated first by comparing experimental data to simulations, then by reconstructing an inclusion in biological tissues.

## Specifications table:

**Table:** 

**Hardware name**	*EIT*_*Hardware*_*16E*	

Subject area	• Electrical Impedance Tomography	
	• Biological sciences	
Hardware type	• Imaging electrical properties of tissues	
	• Injecting currents, measuring voltages, multiplexing	
Cost of hardware	About 250 €	
Additional equipment required to perform EIT	• An Analog to Digital converter 14-bit minimum and 20 digital signals 0 V/5 V (with a Raspberry Pi for example)	
	• A PC	
	• A low frequency generator	
	• A ±15 V power supply	
	• Wires/cables	
	• A body with electrodes to be imaged	

Source file repository	https://doi.org/10.5281/zenodo.5821831	

## 1 Hardware in context

Electrical impedance tomography (EIT) is an emerging technique that has been undergoing strong development over the past twenty years. Using low amplitude AC signals injected through electrodes, a mapping of the admittivity of a medium can be obtained with EIT. This technique is partularly useful to determine properties of human tissues. Common medical applications are then lung[1], brain[2] or even wrist[3] imaging. EIT is appreciated for its portable nature and relatively low cost compared to other imaging techniques. More over, low amplitude electrical signals are harmless unlike X-rays for example. However, EIT has a lower spatial resolution than some imaging techniques such as MRI and CT. In addition, the reconstruction is particularly sensitive to disturbances due to the ’ill-posed’ problem. A high accuracy of the hardware measurement system is then required.

In EIT, the test body to be imaged (commonly referred to as Ω) is generally surrounded by electrodes regularly distributed at the boundaries (commonly referred to as ∂Ω) which serve to apply and measure electrical signals. In order to obtain relevant information for the reconstruction of the entire domain, a large number of measurements are performed, using a 4-pole measurement technique. The principle consists in applying a constant current intensity through the body between two electrodes, then sequentially measuring the resulting voltage between other pairs. This operation is then repeated for different current injection pairs. Measurement data are then post-processed to solve the so-called ‘inverse’ problem, which consists in reconstructing the admittivity map from the measured voltages. Reconstruction can be absolute using a single set of measurements, or differential using a set of reference measurements, commonly obtained by numerical simulation or using an homogeneous medium. This second solution is generally preferred for its higher robustness to modeling errors.

Mapping tissue admittivity by impedance tomography requires significant development at both hardware and software levels. The software part generally includes a finite element meshing, regularization tools used to manage the ‘ill-posed’ character of the problem, and complex minimization tools. A powerful and open-source resolution software commonly used for resolution of the direct and inverse problems is EIDORS[4]. Several EIT systems are commercially available[5], [6]. However, their cost (often several thousand euros) is significant, and they cannot be adapted for specific application. Electronic boards such as STEMLAB of RedPitaya[7] are interesting to implement an EIT system. These electronic boards include tools for the generation, measurement and processing of electrical signals. Their cost (a few hundreds of euros) however remains high, and extra hardware is required to address each electrode independently. Some authors proposed in previous papers EIT hardware designs. In [8], a low-cost and high speed hardware is proposed. Sadly, schematics are not provided. Earlier research works[9], [10] provide hardware schematics and tests of a fully functional hardware. Nevertheless, the method of filtering and demodulating the signals requires the use of a large number of electronic components, which makes the structure relatively bulky. It therefore seems that availability of an efficient hardware at a reasonable cost would be interesting especially for research laboratories where concept validation does not require the level of accuracy of professional but expensive devices.

In this paper, an open-source hardware design, fully-documented, is proposed. Schematics are provided, and can thus be easily reused, modified or adapted. Hardware design choices have been driven to facilitate assembly and to minimize number of components while maintaining measurement quality, which are essential for a quality reconstruction due to the ’ill-posed’ nature of the problem. In particular, signal filtering and demodulation are both performed thanks to a ‘lock-in amplifier’ circuit, thus providing an efficient but compact solution. The procedures for manufacturing and using the hardware are explicitely given so that any user can perform measurements easily. Finally, each part of this hardware is tested to ensure its reliability and determine its characteristics and limitations.

## 2 Hardware description

### 2.1 General overview

**Fig. 1:**
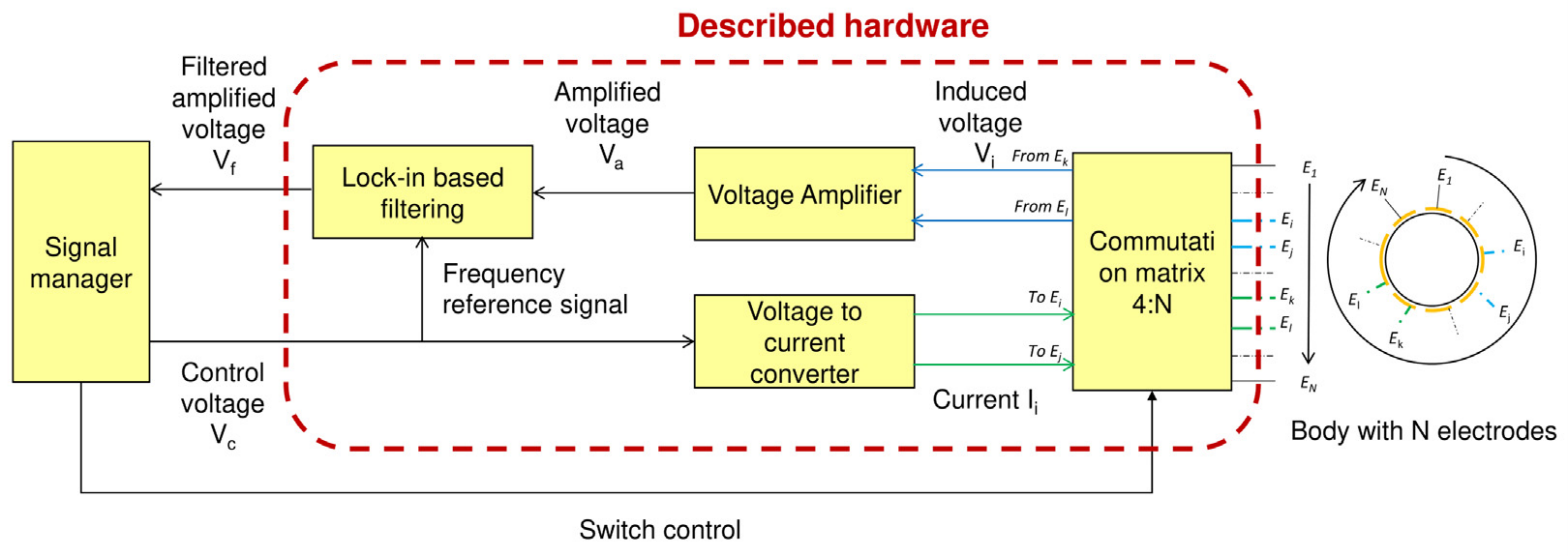
Hardware architecture. A control voltage allows generating a current which is injected in the body through a commutation matrix. This matrix allows selecting a second pair of electrodes, which voltage is amplified and filtered before being driven to the signal manager.

In this section, a general overview of the developed platform is given (see Fig. 1). Each part of the platform is then described individually.

The hardware consists of 4 circuits:

• A Voltage-to-Current-Converter (VCC), or Voltage-Controlled Current Source (VCCS),
• A commutation matrix 4:*N*, in the present case with *N* = 16. The four inputs are connected to the electronics and the *N* outputs to the body’s electrodes.
• A voltage amplifier,
• A Lock-in based filter.

As inputs and outputs of the system are generated and measured voltages, these functions can be performed by commonly available instruments in the lab. Consequently, this paper emphasizes signal routing and analog processing. The term signalmanager will thus be used to refer to generating, measuring and processing devices.

The most common way to perform EIT in a body surrounded by electrodes consists in selecting a pair of electrodes to inject an AC current inside the body using a VCC, and another pair to measure the induced voltage. The operation is repeated with different combinations of injecting and measuring pairs, which provides the information required to build the admittivity map. Pairs are selected using a commutation matrix composed of several multiplexers and demultiplexers. This method, called ‘4-point’ or even ‘4-pole’ sensing, allows reducing the effect of contact impedance between the electrodes and the medium[11]. Yet, voltages that have to be measured are of lower amplitude than for the 2-pole sensing, where the same two electrodes are used to both inject current and measure induced voltage. Signals are thus difficult to measure in 4-pole sensing, and can be mixed with significant level of noise. A voltage amplifier associated with a filter allows raising the voltage to a measurable level, while rejecting measurement noise. A lock-in filter is chosen for its very narrow band filtering capability, its easy implementation using modern commercially available components, and at last for its DC output which can be easily characterized. Additionally, this part facilitates 2-pole sensing by rejecting the tissue/electrode contact capacitive effects. These many features make this part an efficient tool for processing the signal of interest with a reduced number of components. The proposed hardware was designed to generate and handle frequencies from 1 kHz to 50 kHz and sinusoidal amplitudes from 0 to 2 mA signals, i.e. for most EIT applications, but minor modifications, mentioned in the development section, would allow to increase these values.

### 2.2 Voltage-to-current converter

**Fig. 2:**
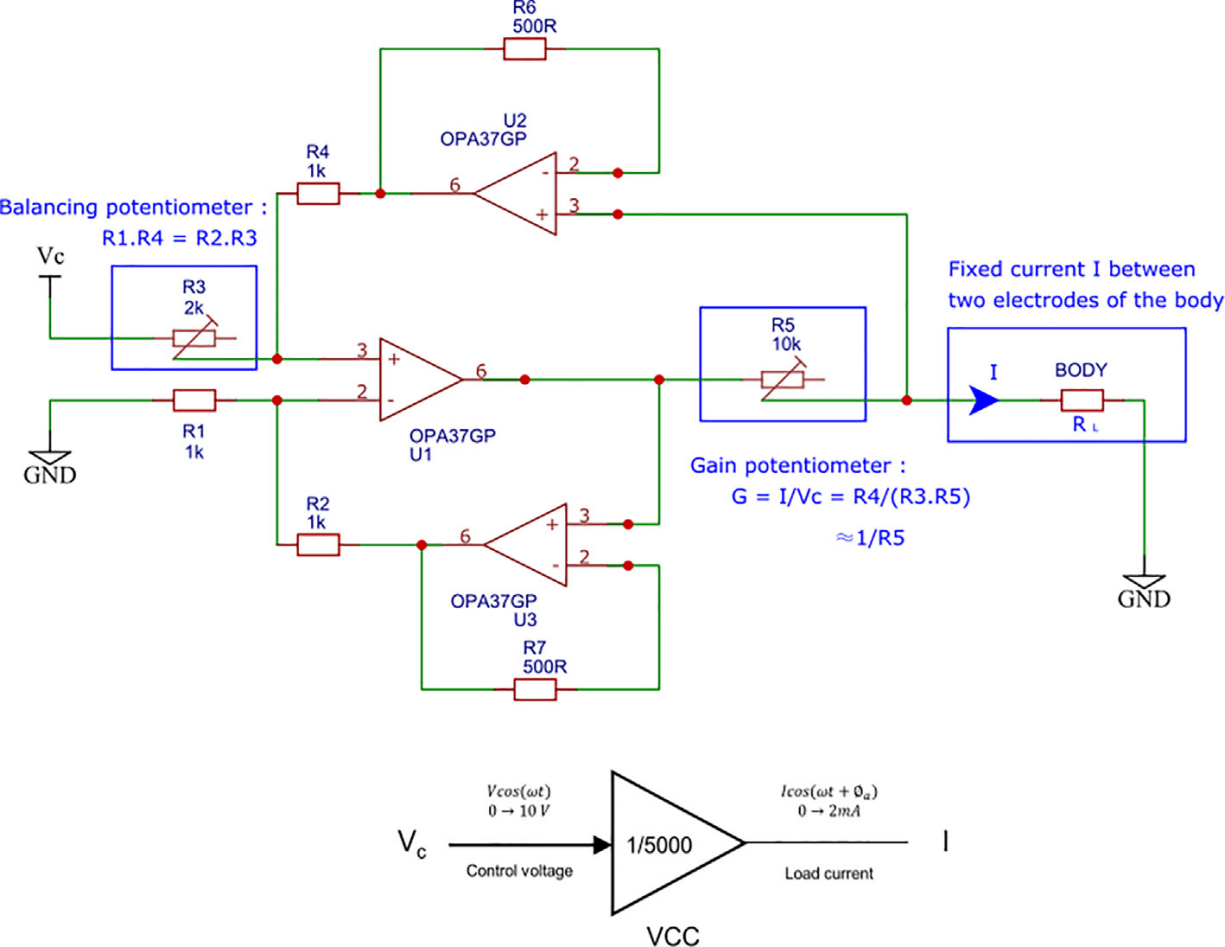
Schematics and high level model of the voltage-to-current converter used in this work and proposed in[12]. This part allows to get a fixed output current for given input control voltage. A potentiometer $R_{3}$ is used to balance the resistors. Another potentiometer $R_{5}$ allows to fix the gain of the system.

In this work, a VCC design with several operational amplifiers (OPA) based on previous works[12] was selected (3-OPA Howland current source). This choice was made after study of its performances and simplicity to implement, as it is described below. A full description of this part can be found in[12], and a comparative study with other architectures were also carried out in [10]. A schematic of the VCC is given in Fig. 2. The circuit must satisfy the equation $R_{1}\;R_{4}\;=\;R_{2}\;R_{3}$ to make the output current *I* insensitive to the load $R_{L}$. Due to inacurracies on common components’s resistance value, $R_{3}$ is a balancing potentiometer which is tuned to satisfy the equation. The following relation is thus obtained:

(1) $$I=\frac{R_{4}}{R_{3}\;R_{5}}V_{c}$$

A gain potentiometer, $R_{5}$, allows to fix the desired gain. In our example, $R_{5}$ is set to obtain *G*  *=* *1/5000* A/V. These tunings allow to convert the input control voltage *V*_*c*_ of the VCC from 0 to 10 V to an output current intensity *I* from 0 to 2 mA.

**Fig. 3:**
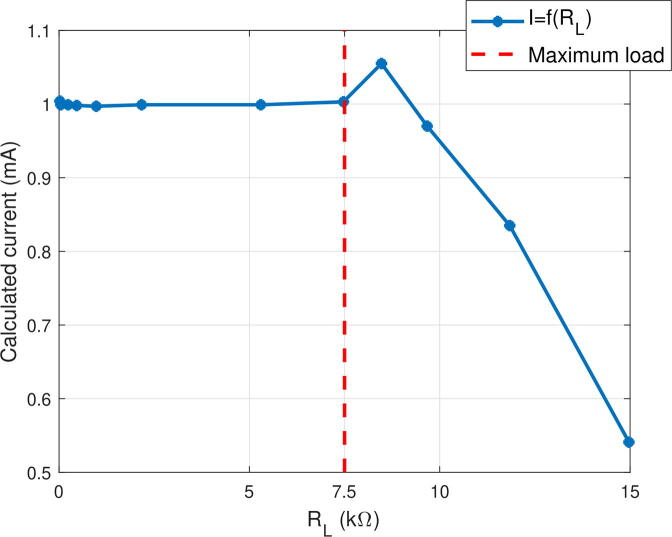
Calculated current amplitude as a function of the load resistance. The current amplitude for a fixed input control voltage generated by the VCC was calculated by measuring the induced voltage passing through a load resistance with a known value.

As resistances of the VCC cannot be manually adjusted with a high precision, the VCC is tested. To do so, load resistances with known values are used to calculate the actual current amplitude. In this test, the VCC injects a current in a load resistance with a known value, and the induced voltage is measured, allowing to calculate the actual current. The amplitude of the input control voltage *V*_*c*_ of the VCC is set to 5 V with a frequncy of 1 kHz, so the current amplitude passing through a load is theoretically 1 mA (gain *G* =1/5000). The value of several load resistors in a range of 21.8 Ω to 14970 Ω is measured beforehand using a multimeter (0.3 % precision multimeter according to the datasheet). Current amplitude is calculated using Ohm’s law, and the curve presenting the calculated current amplitude as a function of the load is given in Fig. 3. Tuning the VCC consists in two steps. In the first step, the potentiometer $R_{3}$ is tuned so that $R_{1}.R_{4}\;=\;R_{2}.R_{3}$ and $R_{5}$ tuned so that $G=1/5000=R_{4}/R_{3}.R_{5}$. Although this setting is theoretically correct, other sources of unbalance in the assembly may exist (offsets of OPAs mounted as a follower, different temperature coefficients of resistors, etc.). Thus, the current intensity-load resistance curve I = f(R) may have a slight slope, and a second adjustment is necessary. In the second step, $R_{3}$ is adjusted after observing the curve so that the current amplitude is constant in the range of 21.8 to 7500 Ω. Finally, $R_{5}$ is also re-adjusted to fix the gain to *G* = 1/5000. The curve indicates that the device presents an error lower than the multimeter resolution if the load does not exceed 7.5 kΩ. Beyond this load value, some signals are saturated in the circuit, and the VCC is no longer able to supply the requested current. The bandwidth of the VCC decreases as the load increases. The value retained as being the bandwidth of the VCC is therefore the bandwidth characterized experimentally for the maximum load (i.e. 7.5 kΩ), whose value is 50 kHz.

To conclude, this VCC presents good performances for many EIT applications despite manual resistance balancing, for loads between 20 Ω and 7.5 kΩ. Error then rapidly increases, which makes this VCC unsuitable for higher loads (more than 7.5 kΩ). Potentiometers were chosen here for simplicity, but for an industrial implementation, digitally-programmable resistances would be preferable. The bandwidth of the VCC is 50 kHz for the maximum load, which is sufficient for most EIT applications. It is possible to increase this value by using OPAs with higher bandwidth-gain product and increasing the output impedance of the VCC, for example with the use of a generalized impedance converter[13].

### 2.3 Commutation matrix

**Fig. 4:**
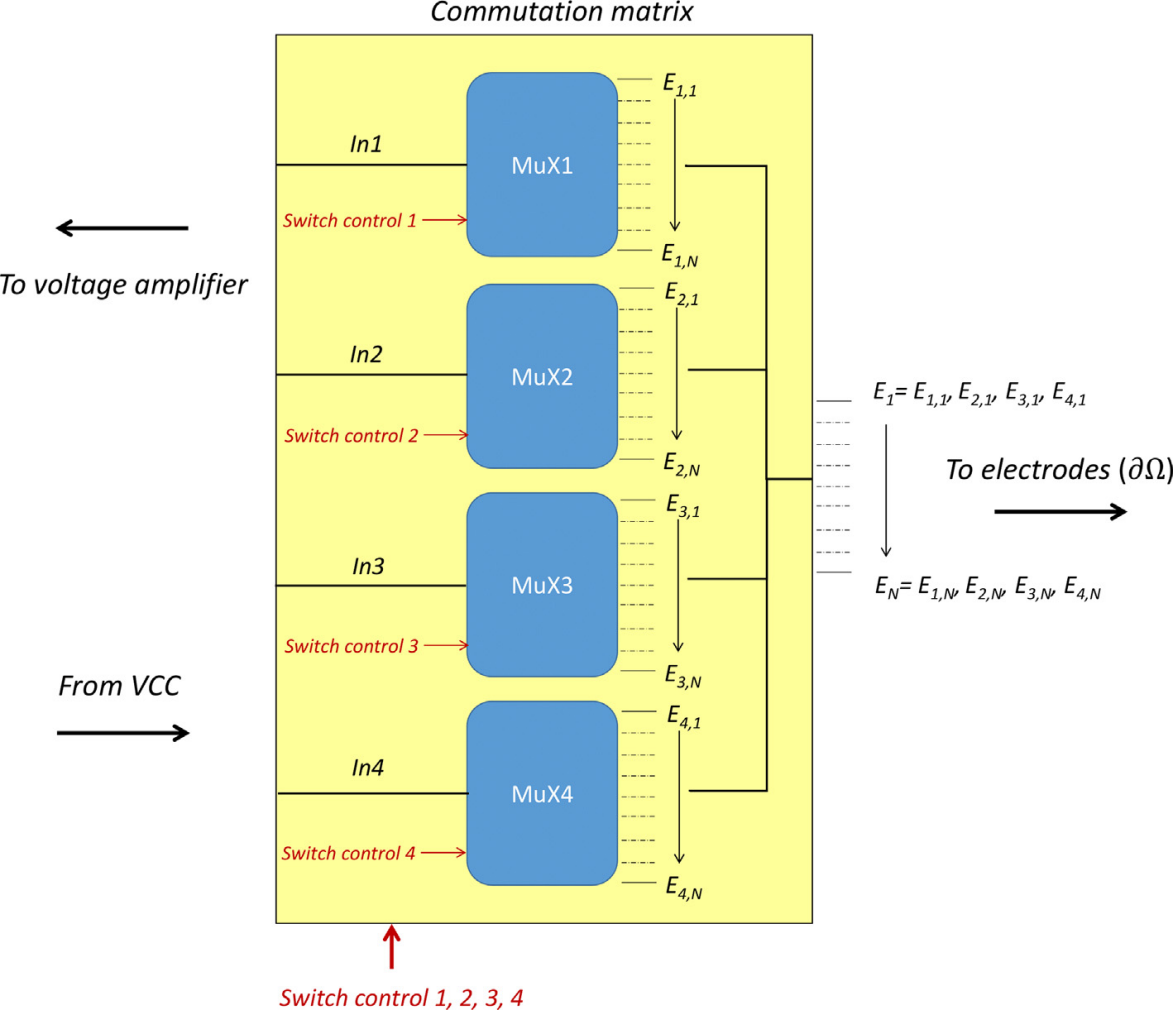
Simplified diagram describing the commutation matrix. Multiplexers are used to adress 4 among *N* electrodes for current injection and voltage measurement.

The system described here is similar to the one developed in [10]. A commutation matrix is a system allowing a number of connections to be made between several inputs and several outputs. In our particular case, the matrix is made of four inputs. Two inputs allow to carry the current between the injecting electrodes, and two other ones allow to carry the resulting differential voltage between the measuring electrodes. A simplified diagram describing the internal structure of the matrix is given in Fig. 4. More details are given in the schematics in supplementary materials.

Four 1:16 multiplexers ADG506AKRZ are used to route signals. These components were chosen for their low noise, high supply voltage and their DIP (Dual Inline Package) type which makes them easy to assemble. The four ‘inputs’ of the matrix for injection and measurement are connected to the inputs of the multiplexers named *In1, …, In4* on the diagram. Channels 1 to 16 of each multiplexer are respectively connected to channels 1 to 16 of the other three so that each ‘input’ has the possibility of being connected to each electrode of the body. Binary codes detailed in the component datasheet allow selection of the output channel. The time required for switching is less than one microsecond and is therefore considered negligible. Like any multiplexer, the ADG506A owns an internal resistance $R_{\mathrm{ON}}$ that has to be considered. In our tests, $R_{\mathrm{ON}}$ is of approximately 150 Ω, and only depends on the multiplexer. No frequency or channel number dependency were noticed. It is wise to measure and add $R_{\mathrm{ON}}$ to the contact impedance model. Although, these resistances do not significantly impact measurements in 4-pole sensing, provided that they are low compared to the input impedance of the amplification stage[14]. In 2-pole sensing, they must be considered. At last, $R_{\mathrm{ON}}$ has no impact on the current amplitude supplied by the VCC as long as the total load does not exceed the maximum load. Each multiplexer is controlled using binary codes. 4 bits are necessary to adress the 16 channels of one multiplexer. 1 bit is also necessary to enable or disable the device. Thus, 20 bits are necessary to control the commutation matrix. It should be noted that it would be possible to reduce this number to 16, but the activation bits here also form part of the control signals in order to be able to deactivate the multiplexers in the safety program. An algorithm was made so that the outputs of two multiplexers cannot be simultaneously connected to the same electrode.

### 2.4 Voltage amplifier

**Fig. 5:**
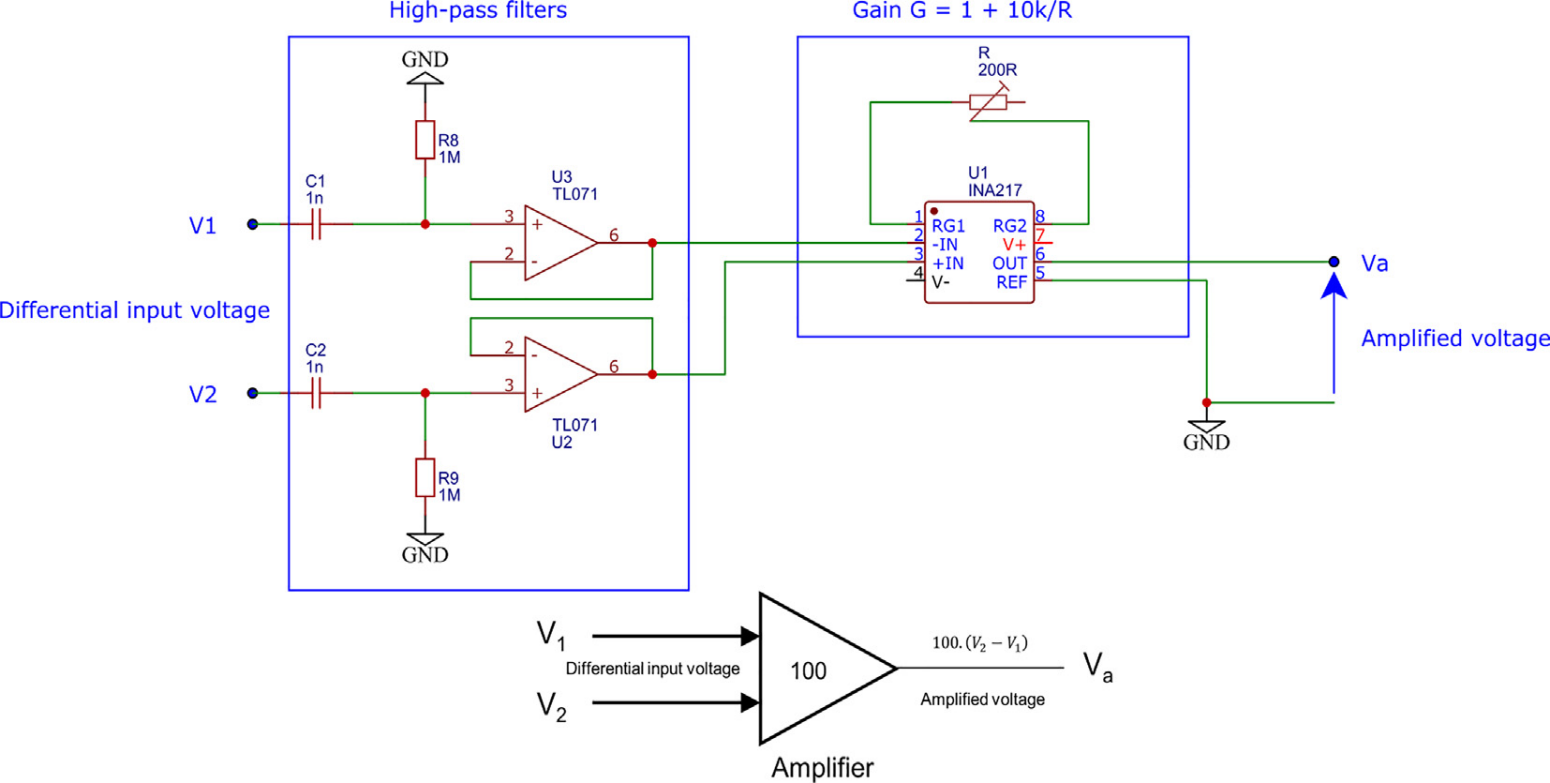
Schematics and high level model of the voltage amplifier. An amplification stage with a gain of 100 multiplies the differential voltage to reach a referenced measurable level.

In 4-pole sensing, measured voltages can be very low (a few mV). Consequently, a voltage amplifier, usually named instrumentation amplifier (INA), is inserted in order to reach measurable voltages. This allows even common ADCs (Analog to Digital Converters), such as Arduino’s (4.9 mV resolution with a standard setup), to be used in order to measure the differential voltage once amplified with a good precision. Noise being amplified as much as the signal of interest, this part is preferentially inserted between the commutation matrix and the filtering circuit. INAs are commonly designed to have interesting intrinsic characteristics in terms of closed loop gain, common mode rejection ratio and input impedance. An INA is generally made with 3 OPAs. Here, the integrated circuit INA217AIP, which is simple to implement and relatively low-cost, is chosen. Schematics of the amplifier are given in Fig. 5. The amplification stage includes two components: an INA217 and a resistance *R* that tunes the gain *G* with the relation $G=1+\frac{10000}{R}$. A gain 100 is chosen for this specific hardware. The bandwith is then 800 kHz, which is consistent with the bandwidth of the VCC (50 kHz). The gain is tuned by adjusting a potentiometer resistance. A high-pass filter is inserted on each input channel to remove DC noise eventually present and which could potentially saturate the INA. Their cutoff frequency in about 150 Hz, which implies using signals with a frequency of at least 1 kHz for negligible attenuation. In a later version, the gain adjustment could be done using a programmable resistor. This would allow tuning the gain depending on the application, or quickly switching between 2-pole and 4-pole sensing.

### 2.5 Voltage filtering and measurement

In order to perform both efficient noise filtering and signal demodulation, a lock-in amplifier, commonly used in EIS (Electrical Impedance Spectroscopy), is exploited. Lock-in technique is used to separate narrow-band signal from wide-band noise. It acts as a detector and a narrow-band filter combined. Using a fixed reference frequency, it is possible to recover the part of the signal at this frequency, even drowned in wide-band noise which power is several orders of magnitude higher. Lock-in based filtering presents several points of interest:

• It is a very narrow-band filter: a reference signal is used and all frequencies different from that of the reference are rejected,
• The control voltage *V*_*c*_ that drives the VCC can be used as reference, and thus no additional signal is required,
• Its output is a DC signal, that can be measured with common equipment,
• The real and imaginary parts of the input signal can be dissociated, which allows for example to reject the capacitive contact impedance between the electrodes and the medium.

**Fig. 6:**
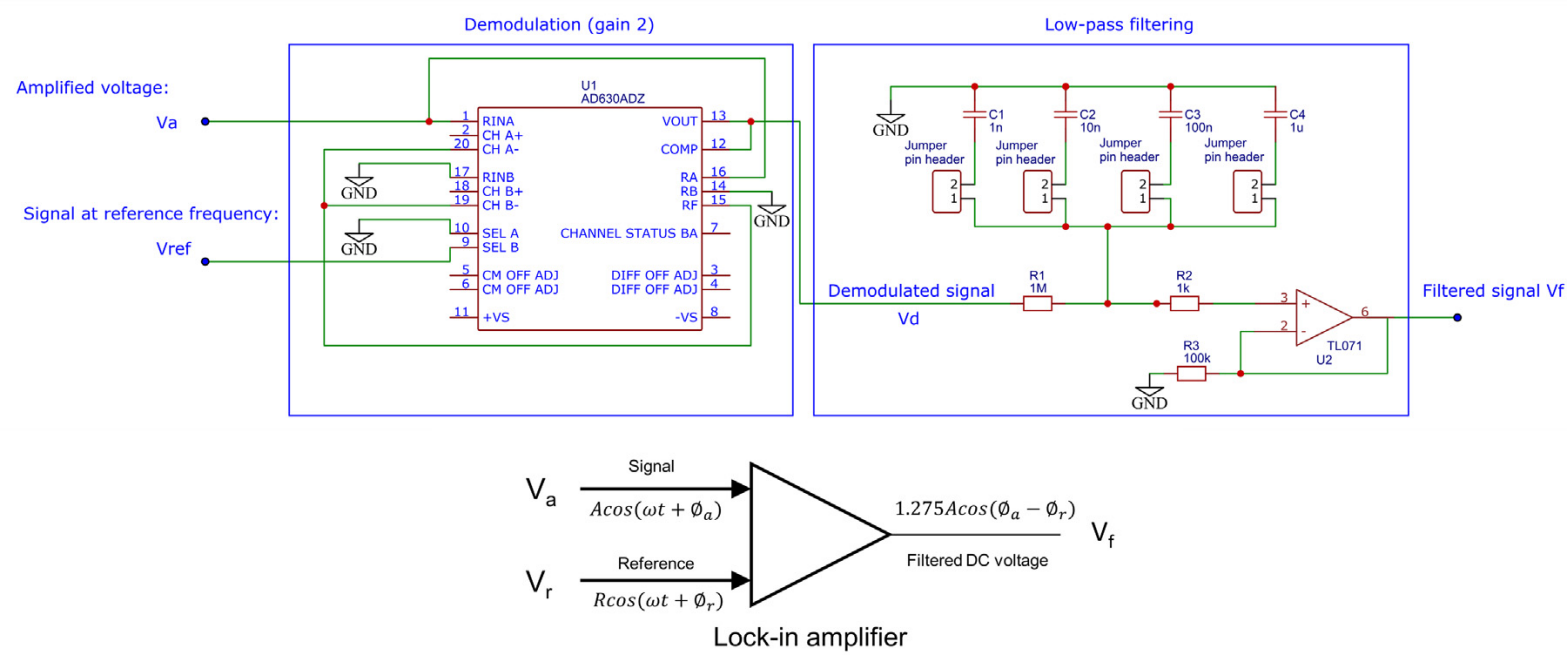
Schematics and high level model of the demodulation/filtering circuit. A demodulation integrated circuit multiplies the input signal $V_{a}$ by a reference $V_{r}$. After low-pass filtering, the output signal is a DC voltage $V_{f}$ proportionnal to the input amplitude and cosine of the difference of phase shift between $V_{a}$ and $V_{r}$.

The proposed circuit, designed using the AD630ADZ integrated circuit, was adapted from[15]. Schematics and high level model of the lock-in amplifier circuit are given in Fig. 6. The first stage is a demodulation circuit, and the second one is a low-pass filter. The principle of the circuit is briefly described below, assuming sine-wave signals. Considering the input signal $V{}_{a}(t)=A\;\cos(\omega_{a}t+\phi_{a})$ and a reference signal $V{}_{r}(t)=R\;\cos(\omega_{r}t+\phi_{r})$, demodulation consists in multiplying these signals to obtain an output voltage $V_{d}(t)$:

(2) $$V_{d}(t)=k\;V_{a}(t)\;V_{r}(t)=k\;A\;R\;\cos(\omega_{a}t+\phi_{a})\;\cos(\omega_{r}t+\phi_{r}),$$

with *k* a constant in V^−1^ which depends on the integrated circuit used and $V_{d}(t)$ the output voltage of the demodulator. From this equation, the demodulator output voltage is:

(3) $$V_{d}(t)=k\;\frac{\mathrm{AR}}{2}\;\left[\;\cos((\omega_{a}-\omega_{r})t+\phi_{a}-\phi_{r})\right)\left(+\;\cos((\omega_{a}+\omega_{r})t+\phi_{a}+\phi_{r})\right]$$

A low-pass filter then averages this voltage:

(4) $$V_{f}(t)=V_{d}(t)=\langle k\;\frac{\mathrm{AR}}{2}\;[\cos((\omega_{a}-\omega_{r})t+\phi_{a}-\phi_{r})+\;\cos((\omega_{a}+\omega_{r})t+\phi_{a}+\phi_{r})]\rangle$$

If $\omega_{a}\;\neq \;\omega_{r}$, then the averaged output voltage $V_{f}(t)$ tends to 0 after a sufficiently long integration time. If $\omega_{a}\;=\;\omega_{r},V_{f}(t)$ is proportional to the input amplitudes and the cosine of the phase shift difference: $V_{f}(t)=k\;\frac{\mathrm{AR}}{2}\;\cos(\phi_{a}-\phi_{r})$. Thus, only information about signals at the reference frequency are detected by the system and any other frequency is rejected. More details about lock-in amplifier applications, noise rejection and principle of functioning can be found in the litterature[16], [17].

Concerning the demodulator, its gain is set to 2. This gain was chosen because of the bandwidth gain product of the AD630ADZ (2 MHz), which gives a bandwidth for this part of at least 1 MHz, and therefore is consistent with the bandwidth of the amplification stage (800 kHz) and VCC (50 kHz). The low-pass filter is an OPA-based first-order filter. Several capacitors can be picked using jumpers. The time constant is τ = *R1.C* with *C*  = *C1*, *C2*, *C3* or *C4* depending on the jumper position. τ can here be tuned from 1 ms to 1 s. A higher order filter could eventually be used for greater attenuation.

The average output DC voltage $V_{f}(t)$ of the lock-in amplifier is independant of the reference signal amplitude and waveform due to the intrinsic properties of AD630ADZ. $V_{f}(t)$ only depends on the signal amplitude *A* and the cosine of the phase shift difference $\cos(\phi_{a}-\phi_{r}),i.e$. on the so-called ‘in-phase’ part of the signal. In this application, the DC output, assuming a sine input wave $V_{a}(t)$, was characterized experimentally:

(5) $$V_{f}(t)=G\;A\;\mathrm{mean}(|\cos(\omega_{a}t)|)\;\cos(\phi_{a}-\phi_{r})\approx 1.275\;A\;\cos(\phi_{a}-\phi_{r})$$

In a later version, a phase shifter could be used to obtain a 90° phase shifted reference signal, and thus would allow to measure the so-called ‘quadrature’ signal. It would then be possible to evaluate the impedance module and phase angle.

## 3 Design files summary

.

**Table:** 

**Design filename**	**File type**	**Open source license**	**Location of the file**

*EITHardware16E*_*EasyEDA.zip*	*CAD file*	*EasyEDA*	https://doi.org/10.5281/zenodo.5821831
*EITHardware16E*_*Gerber.zip*	*Gerber file*	*-*	https://doi.org/10.5281/zenodo.5821831
*EITHardware16E*_*Schematics.pdf*	*Schematics*	*-*	https://doi.org/10.5281/zenodo.5821831

The file *EITHardware16E*_*EasyEDA.zip* contains the full schematics in the EasyEDA format. The platform consists of all the parts described above, assembled together. It is possible from the EasyEAD software to convert the file into another format (Altium,…). The file *EITHardware16E*_*Gerber.zip* allows fabrication of the PCB. The file *EITHardware16E*_*Schematics.pdf* contains a pdf version of the schematics. Contrary to the simplified schematics given in the paper, this document includes all components (for power supply,…).

## 4 Bill of materials

The bill of materials provided in supplementary material can be used to get the required components. It is advised to get additional active components.

## 5 Build instructions

Making and calibrating the hardware requires the following steps:

1. Use the gerber file EITHardware16E_Gerber.zip to order the PCB
2. Use the bill of materials to get the required components
3. Solder all components, including winslows for integrated circuits. See schematics in *EITHardware16E*_*Schematics.pdf* for details
4. Tune the VCC potentiometer (balancing) as described in Section 2.2
5. Tune the amplifier potentiometer(s) (gain(s)) as described in Section 2.4
6. Place jumpers: between each part to connect them, to a capacitor at the low-pass filter to adjust its time constant, at the amplifier to select one or two amplification stages

## 6 Operation procedure

**Fig. 7:**
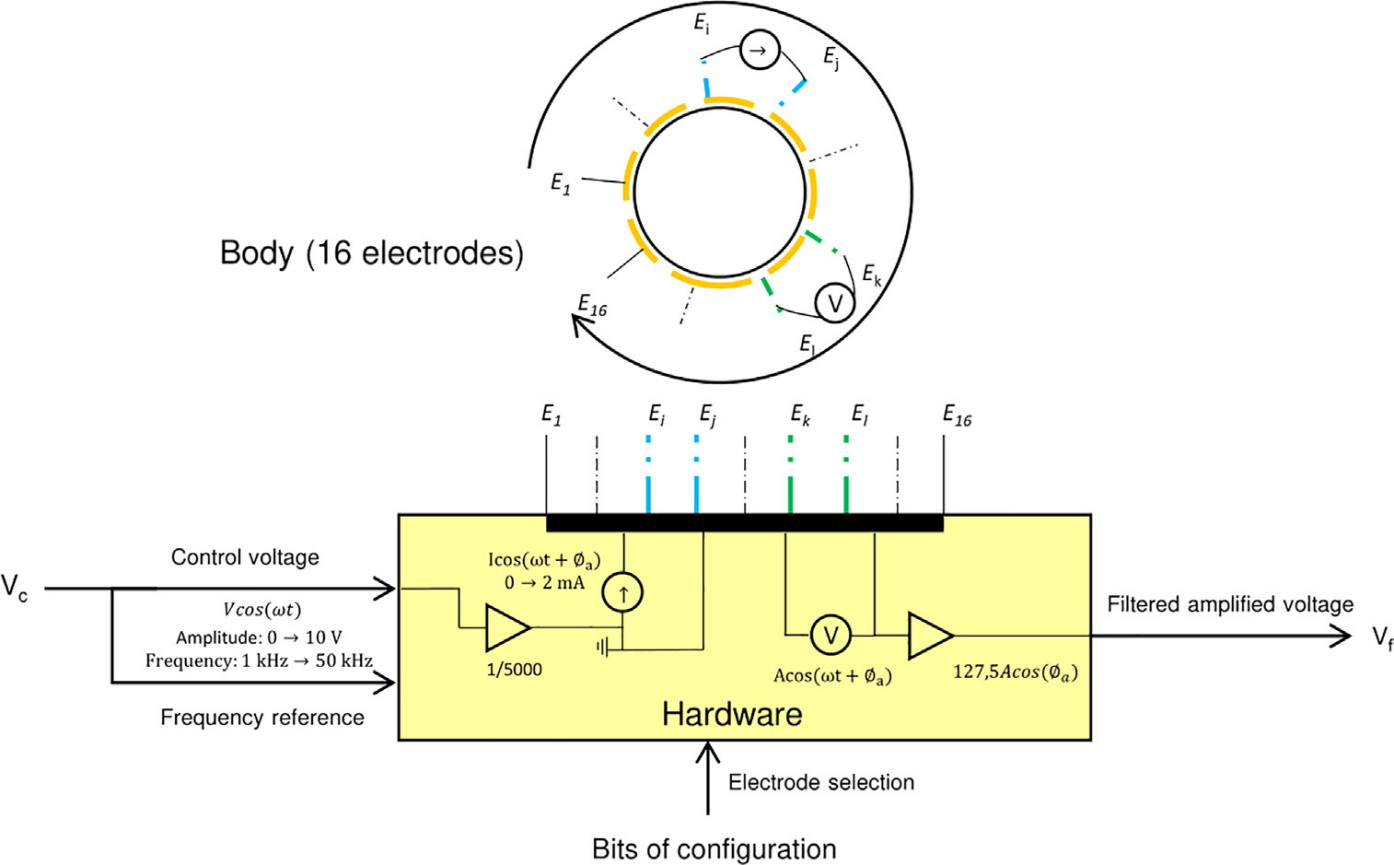
Simplified diagram presenting the global functioning of the proposed hardware.

The four parts of the proposed hardware were described previously. A summary of the hardware operation with these parts is given in Fig. 7. The VCC is based on previous works, and is a 3 OPA structure. This part can provide a constant current amplitude, for resistive loads below 7.5 kΩ, from 0 to 2 mA and from 1 kHz to 50 kHz, which is satisfying for many EIT applications. A commutation matrix was proposed. This part is designed with two ‘inputs’ for current injection, two ‘inputs’ for voltage measurement, and 16 outputs for the selection of four electrodes among sixteen. The matrix is driven using binary codes. An instrumentation amplifier with a gain of 100 and good performances was proposed. At last, a powerful filtering tool based on a lock-in amplifier structure was proposed. This part is easy to design, performs a narrow-band filtering, rejects electrode double-layer capacitive effects, and returns a DC signal that can be easily quantified by common equipment available in the lab. The lock-in amplifier gain, i.e. the gain between the DC output and the real part of the input voltage amplitude is 1.275. Thus, the gain between the referenced DC output and the differential input voltage amplitude across the measuring electrodes is equal to 127.5 due to the amplification stage. Using this hardware requires:

• A low frequency generator, providing the voltage reference,
• Numerical signals to control the commutation matrix, 20 voltages being 0 V or 5 V are necessary,
• An ADC (analog to digital converter) or an oscilloscope allowing to measure the DC output voltage,
• A +/- 15 V power supply,
• Integrating the multiplexer internal resistances in the contact impedance model (in 2-pole sensing).

**Fig. 8:**
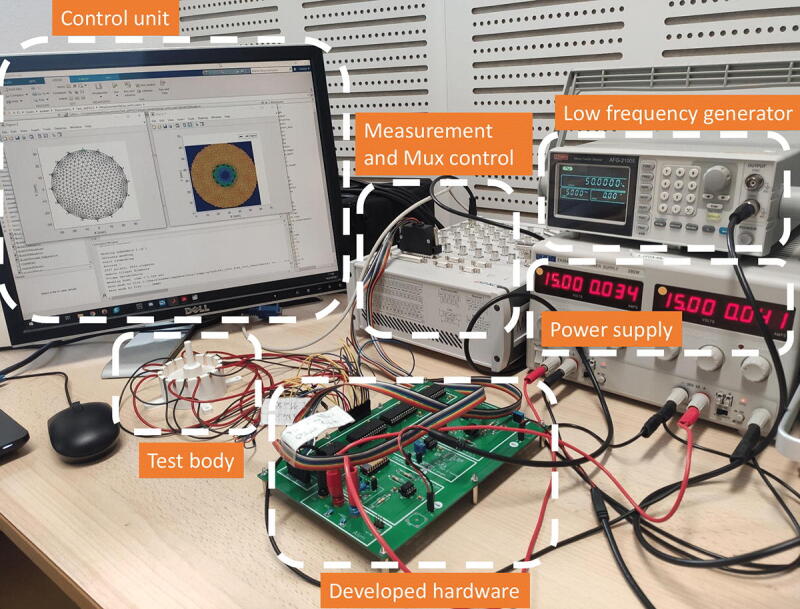
Photograph of the EIT setup, including the developed hardware.

The cited requirements can thus be performed using basic equipment. A photograph of the assembled developed hardware, used within a complete measurement platform comprising a test body and additional instrumentation material, is given in Fig. 8. Using the hardware is done through the following steps:

1. Connect power to the hardware: GND, −15 V, +15 V
2. Connect the low frequency generator to the VCC input
3. Connect the 20 digital signals (0 V/5 V) to the control part of the switch matrix. This can be done with an Arduino mega for example
4. Connect the measuring device to the output of the lock-in amplifier filter. An Arduino may be suitable, but it is advisable to use a device with a 14-bit resolution
5. Plug the body that one wants to image at the ’output’ of the commutation matrix
6. Switch on the power supply
7. Switch on the low frequency generator and the measuring device
8. Tune the signal at the low frequency generator. For example, 5 V at 10 kHz to get 1 mA at 10 kHz at the output of the VCC.
9. Tune the 20 digital signals to select the electrodes. The first ten are used to set electrodes 1 and 2 for current injection, the last ten are used to set electrodes 3 and 4 for measurement. The truth table allowing tuning of the multiplexers is available in their datasheet. The first bit is set to 1 (5 V) to turn on the multiplexer. The four other ones allow to code the electrode index as binary. For example, [5 V, 0 V, 0 V, 5 V, 5 V] will activate the multiplexer and select the 4th electrode, [5 V, 0 V, 0 V, 0 V, 0 V] will activate the multiplexer and select the 1st one,….
10. Wait the signal is in steady state (duration depending on the low-pass filter tuning)
11. Get your measurement and save it
12. Divide your measurement by the gain of the system: 127.5. See Section 6 for details
13. Repeat instructions 9→12 for all the required measurements with different combinations of electrodes

## 7 Validation and characterization

### 7.1 Validation of the proposed hardware

**Fig. 9:**
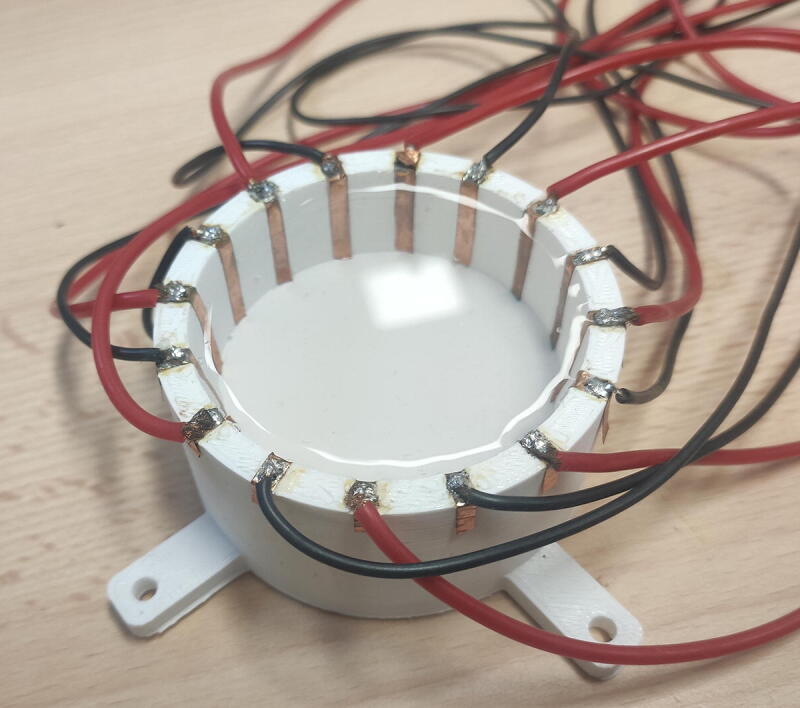
Photograph of the test body. A 60 mm diameter and 30 mm high body made with fast prototyping is surrounded by sixteen 3 mm width copper electrodes made with rustproof copper tape.

In this section, the good functioning of the developed hardware is validated on a common application case: the boundaries are a cylinder surrounded by 16 regularly-spaced electrodes. The body is composed with conductive water of 0.2 S/m. A picture of the body is given in Fig. 9. The cylinder is 60 mm diameter and 30 mm high, electrodes are 3 mm width and 30 mm high. It is proposed here to validate the quality of the hardware in three steps:

1. the voltage set is collected for the homogeneous case with 0.2 S/m saline water,
2. this set of voltages is compared with simulation data,
3. the presence of an inclusion within a biological material is attempted to be reconstructed using the experimental voltage sets obtained from two different configurations (time-difference EIT).

Adjacent pair drive and sensing are performed using the 4-pole measurement technique, which gives 208 measurements. An amplitude of 5 V at 50 kHz is generated from a low frequency generator. This signal is used as control voltage *V*_*c*_ applied to the VCC, to provide a body’s input current with an amplitude of 1 mA, and as frequency reference for the lock-in amplifier. Induced voltages pass through the voltage amplifier and then through the lock-in amplifier which output is a DC voltage $V_{f}(t)$, providing the information about the real part of the impedance. A dSPACE MicroLabBox is used to generate 20 signals from its DI/Os which control the commutation matrix, and to measure $V_{f}(t)$. This device was used because it was available in our laboratory, but this could be done with a standard microcontroller.

**Fig. 10:**
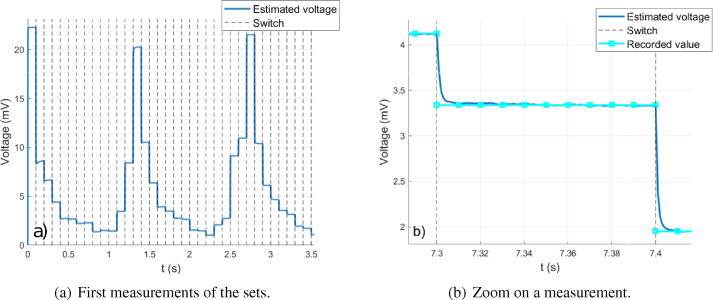
Measured voltage amplitudes before amplification. 100 ms measurements were performed, and 1 ms low-pass filtering used.

**Fig. 11:**
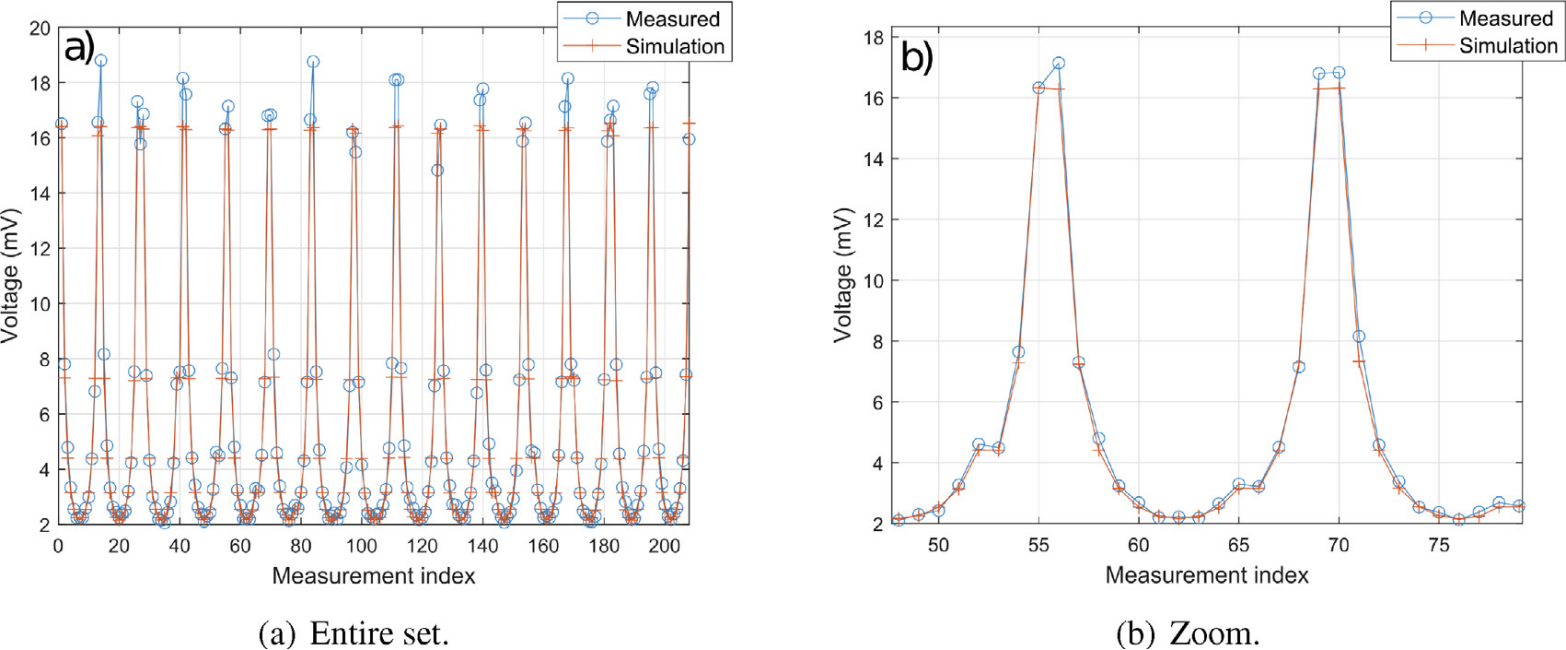
Comparison of the voltages for the homogeneous case at 0.19 S/m, between simulation and experimentation. The recorded voltages for the experimental case are the final values (cyan colored lines in Fig. 10.b).

Measurements must at first be performed. As measured voltage amplitudes are amplified by a gain 127.5 due to the instrumentation amplifier (gain 100) and to the lock-in amplifier (gain 1.275), these values are thus divided by 127.5. A plot of the estimated voltages (i.e. measures once divided by 127.5) is given in Fig. 10. The blue curve represents the estimated voltages for the homogeneous case with saline water. The cyan curve (Fig. 10.b) represents the voltages in steady state which are recorded. The order of magnitude of measurement noise standard deviation in steady state is 10 μV, which is negligible. In order to ensure that the hardware is able to return correct voltage values, measurements are compared to simulations. EIDORS[4] is chosen to perform this task in Matlab. Alternatively, this could be done using the freeware Octave. The body and electrodes are designed and meshed. The input current amplitude is set to 1 mA in EIDORS. Saline water is used, which conductivity is 0.2 S/m at $25^{{}^{\circ}}$C (value given by a conductivity meter). During measurements, the ambient temperature is $23^{{}^{\circ}}$C, so the conductivity of the solution is approximately 0.19 S/m. This value is used in the simulation under EIDORS. A comparison between simulation and experimentation is given in Fig. 11. The voltages measured experimentally are close to simulation’s, which validates the quality of the hardware. The small differences observed can have multiple causes: error on the solution conductivity, on the electrode positioning and width, approximations during the resolution by finite elements, ….

**Fig. 12:**
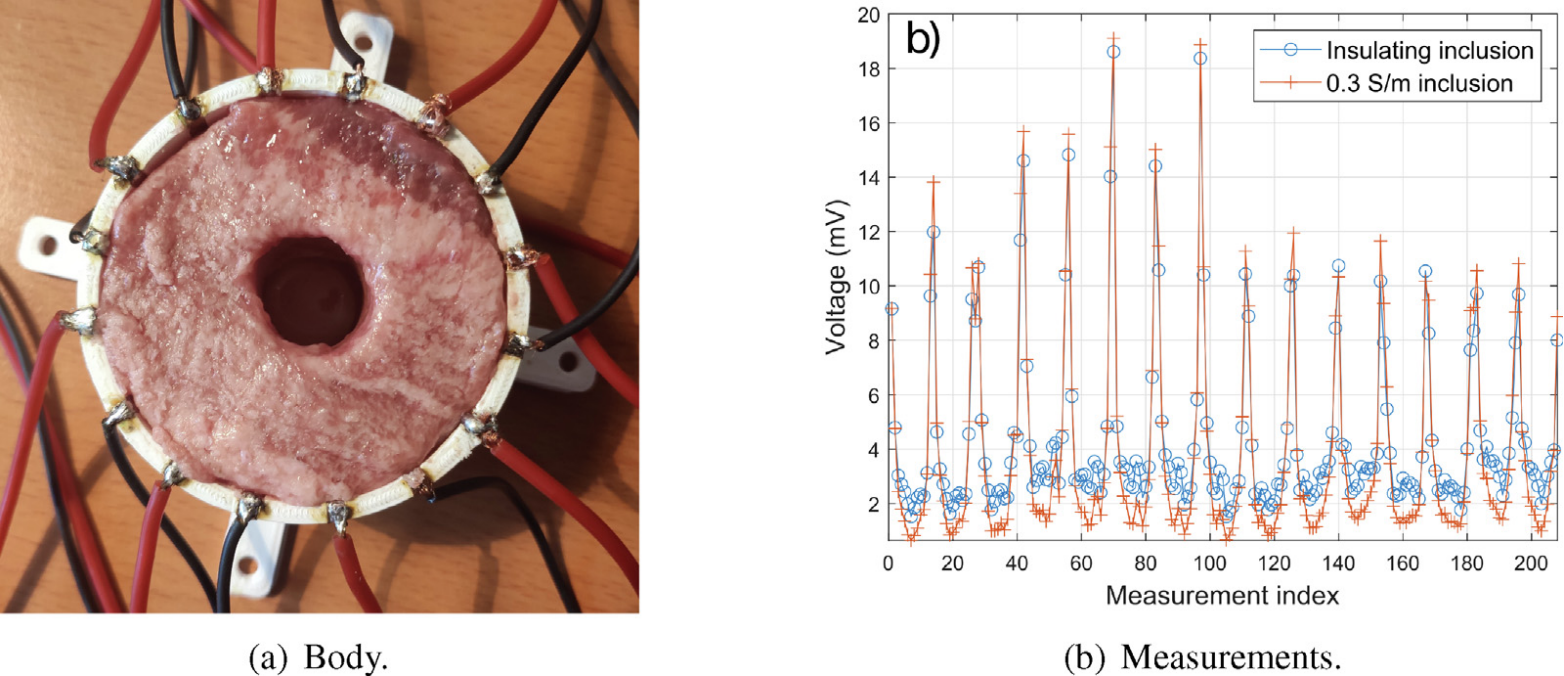
Experimental validation of the proposed hardware using biological tissues. (a) Photograph of the body made bith beef tongue. (b) Measurements using adjacent pair drive for two configurations: insulating (empty) inclusion and 0.3 S/m inclusion.

**Fig. 13:**
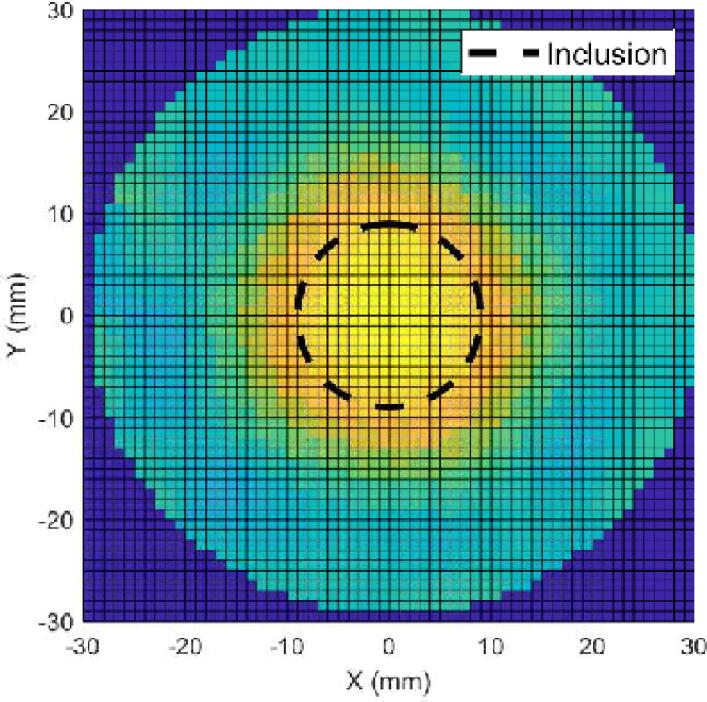
Reconstructed image with Gauss–Newton one-step algorithm and *h*  = 10^−2^.

Now the presence of an inclusion in a biological tissue is attemped to be reconstructed. A cylinder is cut from a beef tongue, frozen beforehand. This cylinder approximately conforms to the shape of the body (30 mm high, 60 mm diameter). Inside, a 18 mm diameter cylinder is cut (see Fig. 12.a). This allows to simulate a temporal variation of conductivity. The objective here is to reconstruct the inclusion using time-difference EIT. To do this, a first set of voltages is acquired, when the inclusion is empty. This is therefore insulating. A second set is acquired by filling the inclusion with a solution of conductivity 0.3 S/m. The voltages are acquired in the same way as before, and plotted in Fig. 12.b. Solving the inverse problem includes two steps: regularization, and minimization. The regularization helps to reduce the ‘ill-posedness’ of the problem. The NOSER (Newton’s One-step Error Reconstructor)[18] algorithm is used to estimate the resistivity on each node of the mesh that minimizes the regularized difference. Only the resistive part is reconstructed. Results are plotted in Fig. 13. As it can be seen on the plot, the inclusion was successfully reconstructed using EIDORS fed by the voltages provided by the proposed hardware. The smoothing effect of the regularization implies that the reconstructed conductivity does not change as abruptly as in reality. Nevertheless, the shape can be easily distinguished, which is fully satisfying. Additional experiments are available in supplementary material.

### 7.2 Characterization

The main characteristics and performance indices of the proposed hardware have been listed in Table 1. The performances were evaluated following the protocol proposed in[19]. It is possible to refer to the document for detailed explanations. Performance measurements are made with the body filled with salt water at 0.19 S/m (visual of measurements given previously in Figure 10).

**Table 1:** Hardware characteristics.

Characteristic	Value	
Dimensions	15 cm × 22 cm × 3 cm	
SNR (mean)	60.6 dB (in steady state with 1 mA injected, 1 ms low-pass filtering at the lock-in amplifier)	
Accuracy (mean)	97 % (some errors may be due to the manual cutting and positioning of the electrodes)	
Acquisition speed	10 ms minimum for each electrode configuration	

**Fig. 14:**
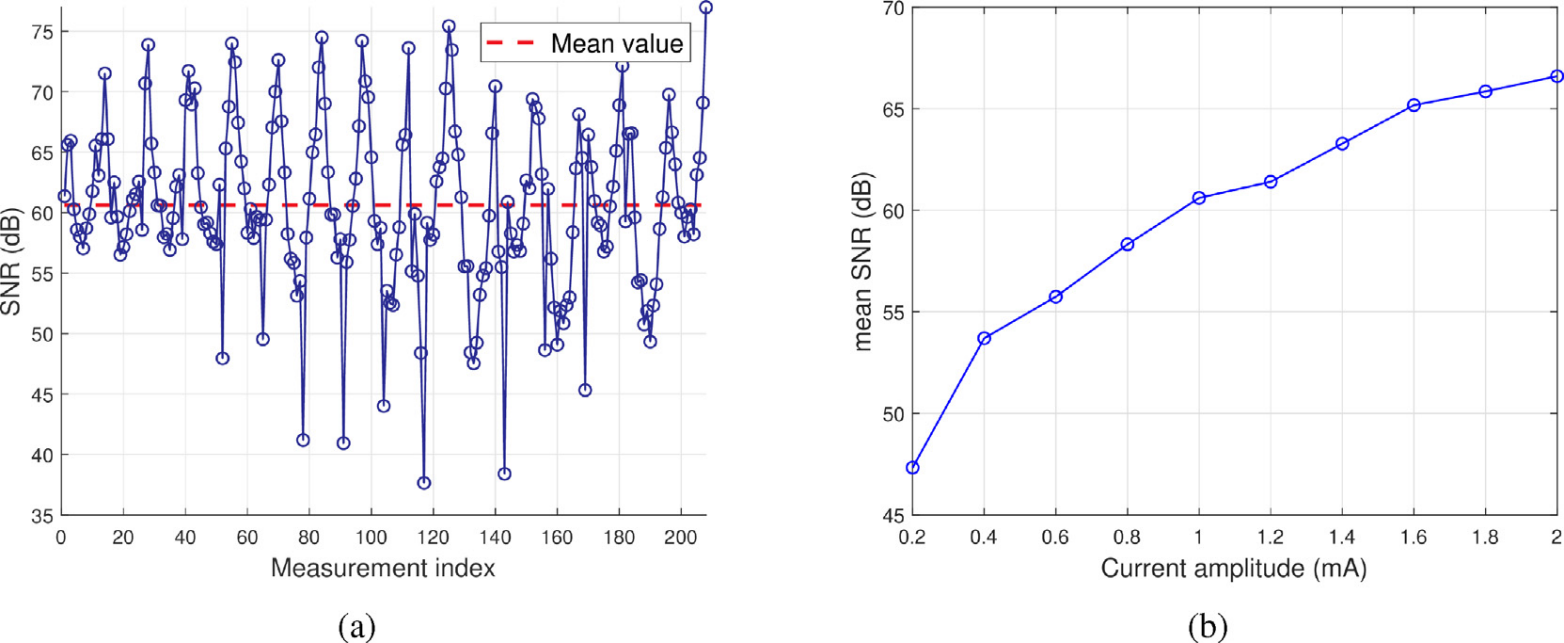
SNR evaluation. (a) SNR for each electrode configuration for the homogeneous case. (b) Mean SNR as a function of current amplitude.

The SNR (signal to noise ratio) is estimated from the ratio of the mean signal to the noise level for each measurement in steady state, *i.e.* after convergence of the lock-in low-pass filter. Experimental parameters were as follows: 1 mA current intensity, low-pass filtering with 1 ms time constant, 14 bit ADC, 100 ms measurements. The data used for the performance calculations are the steady state voltages. Consequently, the performance data for each measurement configuration is taken between t = 50 ms and t = 100 ms. A plot of the SNR for each electrode configuration is given in Figure 14.a. The average SNR is 60.6 dB, which allows us to conclude that noise level is very low. Some exceptional measurements show a lower but still acceptable SNR (35 dB). Choosing a longer low-pass filter integration period, as well as ADCs with a higher resolution would probably increase these values. The variability of the SNR is due, among other causes, to the variability of the amplitude of the measured signals. An adaptive gain at the instrumentation amplifier would allow to homogenize the SNR. In Figure 14.b, the mean SNR as a function of the current amplitude is given.

**Fig. 15:**
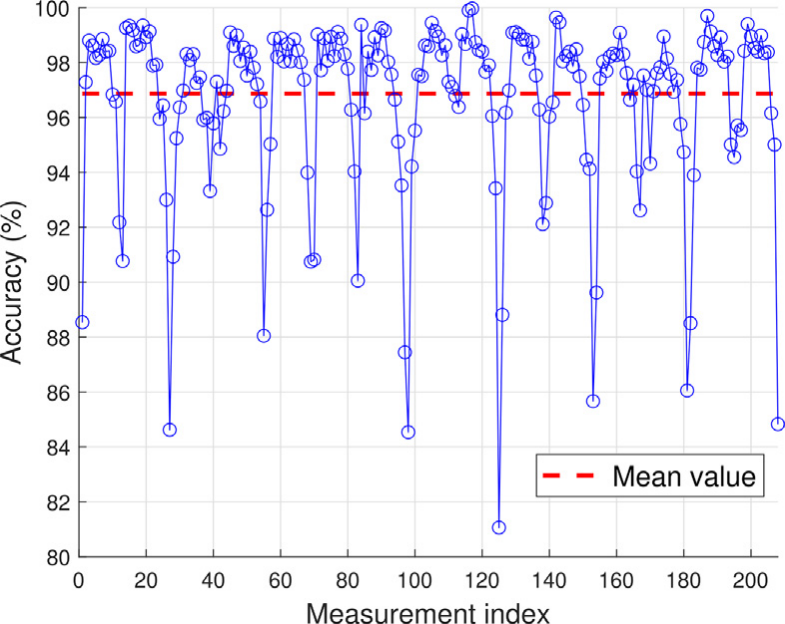
Estimation of the accuracy by comparison between experimental measurements and theoretical data.

Accuracy quantifies the correspondence between theoretical (numerical) and experimental data. As the cutting and positioning of the electrodes is manual, the accuracy is related to the quality of the hardware but also to the model errors that may be present. A plot of the accuracy for each measurement is given in Figure 15, the mean value of which is 97 %.

Since the switching time of the multiplexers is negligible (< 400 ns), and assuming a sufficiently fast multiplexer controller and measurement setup, the decisive element for the maximum acquisition speed that can be reached is the integration period of the low-pass filter at the lock-in amplifier. The choice of the time constant is a compromise between the quality of the filtering and the speed at which the steady state is reached. The integration period must also be large before the period of the signal itself. For this hardware, the minimum time constant of the low pass filter is 1 ms. Considering that the steady state is reached after 10 periods, the acquisition speed is 10 ms per measurement.

## 8 Conclusion and perspectives

The aim of this paper is to describe a precise, low-cost and easy to assemble hardware design for EIT applications. Performance, cost and complexity have driven all the design choices and the so-obtained proposed hardware is relatively simple to use and manufacture while remaining efficient and functional. Overall, the proposed EIT system consists of a current generator, a switching matrix, a voltage amplifier and a lock-in amplifier for filtering. The current source and the switching matrix were already developed in previous works. These devices being relevant with respect to our requirements, we have just used them as they are. Behavior has been explained and discussed, with a specific attention paid to the impedance of multiplexers in the switching matrix, and the accuracy of the generated current with respect to various resistive loads. Overall, current intensity can be considered as constant for typical loads commonly observed in EIT applications at the price of two calibration steps to reach the required level of performance. For voltage amplification, we selected instrumentation amplifiers that are easy to tune and to assemble; these compact devices also present better performances than self-made amplifiers, while remaining available for a fair price. For filtering signals, a lock-in amplifier was chosen for its narrow-band filtering, easy to read DC output and double-layer capacitive effect rejection. Design choices have been made with a specific attention paid to assembly. The proposed solution is then easy to assemble and fully documented to allow open-sourcing, re-use and adaptations. Finally, experimental validation has been conducted on a cylindrical body surrounded by sixteen electrodes. Measurements using the hardware were compared with simulation data with saline water. Also, the presence of an inclusion inside biological tissues were reconstructed using time-difference EIT. The high fidelity of the reconstruction validates the overall quality of the proposed hardware. This hardware has been designed with the aim of achieving both simple design and good performance at low cost. As the design files are shared, the user is free to make modifications to improve performance for specific applications. Several optimizations are possible depending on the desired application. BNC connectors can be implemented in the switch matrix for better signal stability. Digital demodulation could be used to avoid the uncertainties associated with analog components. Automation would allow faster adjustment of the VCC and the voltage amplifier. Finally, a higher order low-pass filter at the lock-in would provide greater attenuation. At last, future works and extensions, to bring compatibility with frequency-difference EIT, will concern the addition of a phase-shifter in the lock-in amplifier to generate a quadrature signal, and thus to calculate both real and imaginary parts of measured signals and to upgrade components to their higher bandwidth versions.

## Declaration of Competing Interest

The authors declare that they have no known competing financial interests or personal relationships that could have appeared to influence the work reported in this paper.
